# The Story of the Insane from Year to Year

**Published:** 1905-09-30

**Authors:** 


### THE STORY OF THE INSANE FROM YEAR TO YEAR.

Seventeenth Annual Series.

### GLASGOW DISTRICT ASYLUM AT GARTLOCH.

At the beginning of the year this Asylum contained 621 patients, and at the end of the year the numbers had increased by 33. There were 156 first admissions; 84 readmissions, 11 unknown whether first or not; 84 recoveries; 68 discharged relieved, and 66 died. The average number resident was 635, and the total number under treatment was 860. The recoveries stand at 33·4 per cent. of the admissions, and the deaths at 10·3 per cent. of the average number resident. The former percentage is lower than the average at Gartloch, and the latter above the average. Of the year's deaths 31 were caused by cerebral and spinal diseases; 3 by pneumonia; 8 by consumption, and 3 by enteritis. Table VIII. shows the causes of the insanity in the admissions of the year, and from it we learn that mental shock and other moral causes account for only 5; while among the 140 in which physical causes were present, alcohol accounts for 42 as the sole cause, and for 11 as contributory. Only 3 are known to have had hereditary tendency. The admissions were 34 fewer than in the previous year, and Dr. Parker thinks this may be owing to the fact "that trade has been bad with the usual result of lessening illness." During the last six years 153 chronic patients have been boarded out, and thus the necessity of further enlarging the asylum has been avoided, for a time at least. Dr. Parker believes, like most medical superintendents north of the Tweed, in boarding out, but he has not lost sight of the disadvantages. Among these he points out the fact that "the residual troublesome cases come into closer and more irritating contact, minus the padding of quiet and orderly folk who have been boarded out, and the proportion of staff per patient increases owing to the average character of the cases left being worse than previously." As already said, there were eight deaths from phthisis, showing that the sanatorium treatment in use at Gartloch has reduced the death rate. Dr. Parker hopes for a still greater reduction; but he is constrained to add "that the lives of these helpless, hopeless, and useless folk will be indefinitely prolonged, thereby adding to the insane population and introducing a new fallacy in the estimation of the apparent growth of insanity." Verily the asylum physician has an anxious time. He knows that almost every patient he discharges recovered or relieved will probably increase the tale of insanity in the next generation, and now he has the consumptive sanatorium on his shoulders, carrying all this weight to lengthen the lives of the tuberculous and the unfit. But duty calls and he must do it. An admirable nursing scheme has been arranged whereby a complete three or four years' course of instruction may be obtained. We welcome this scheme warmly. For years past we have recommended a complete three-years' system in our English asylums, but it has been uphill work, and we believe that the hospital course is confined to the very few asylums which first started it.

